# The arrival of biosimilar monoclonal antibodies in oncology: clinical studies for trastuzumab biosimilars

**DOI:** 10.1038/s41416-019-0480-z

**Published:** 2019-07-01

**Authors:** Liese Barbier, Paul Declerck, Steven Simoens, Patrick Neven, Arnold G. Vulto, Isabelle Huys

**Affiliations:** 1 Department of Pharmaceutical and Pharmacological Sciences, KU Leuven, Leuven, Belgium; 2 Department of Oncology, UZ Leuven, Leuven, Belgium; 3 Hospital Pharmacy, Erasmus University Medical Center, Rotterdam, The Netherlands

**Keywords:** Breast cancer, Antibody therapy, Biological therapy, Breast cancer, Phase III trials

## Abstract

The monoclonal antibody trastuzumab (Herceptin®), which targets the human epidermal growth factor receptor 2 (HER2), is approved for the treatment of early breast and advanced breast and gastric cancer in which HER2 is overexpressed. Several biosimilar versions of trastuzumab are expected to enter the European market over the course of 2018 and 2019. The biosimilar development pathway consists of a comprehensive comparability exercise between the biosimilar candidate and the reference product, primarily focussing on data from analytical studies. Clinical studies for biosimilar candidates follow a different design to those for a new biological, as the aim is not to independently establish clinical benefit, but to confirm biosimilarity between the two agents. The different trastuzumab biosimilar candidates have followed diverse pathways in their clinical development, with differences in clinical trial design (equivalence or non-inferiority design), patient population (those with metastatic or early breast cancer) and endpoint (overall response rate or pathological complete response). These differences in approach in phase 3 testing must be viewed in the totality of evidence demonstrating biosimilarity. Adequate information on the biosimilar approval pathway, the nature of the biosimilarity exercise and how the clinical development of a biosimilar is tailored to meet the licensing requirements can help informed decision making in clinical practice.

## Background

Biological medicines, and anticancer biological medicines in particular,^1^ represent a growing financial burden on healthcare budgets. The loss of exclusivity rights on original biological medicines has allowed biosimilar medicines to enter the market. Biosimilars offer cost-effective treatment options that can help contain the rising healthcare expenditure. The European Medicines Agency (EMA) defines a biosimilar as ‘a biological medicinal product that contains a version of the active substance of an already authorised original biological medicinal product in the European Economic Area’.^2^ Owing to the intrinsic variability that is inherent to all biological medicines, and the complex manufacturing process of these products, a biosimilar cannot be considered an identical copy of the originally approved biological product (the reference product or originator).^3,4^ Minor differences can exist between the biosimilar and the reference product, but it needs to be demonstrated that these differences are not clinically meaningful.^2,3^ ‘Similarity to the reference medicinal product in terms of quality characteristics, biological activity, safety and efficacy based on a comprehensive comparability exercise needs to be established’.^2^ Table 1 provides an overview of the difference between biosimilars and copies of originally approved small-molecule medicines, called generics.

**Table 1:** The difference between biosimilars and generics

A generic is a copy of a an existing small-molecule-based therapeutic and its approval is based on the demonstration of bioequivalence with its reference product by appropriate pharmacokinetic studies.^2,27^
A biosimilar is a biological medicinal product that is highly similar to an already licensed biological medicine, the reference product.^2^ Owing to the intrinsic variability that is inherent to all biological medicines and the complex manufacturing of these medicines, it is impossible to produce identical products. Minor differences can thus exist between the biosimilar and the reference product, however it needs to be demonstrated that these differences are not clinically meaningful.^3^
The development of a biosimilar is based on the demonstration of biosimilarity via extensive head-to-head comparability studies to the reference product.^2^
Generics and biosimilars both follow an abbreviated development pathway for regulatory approval compared with that of an original medicine, however, the requirements are different. As a biosimilar cannot be an exact copy of the reference product, owing to the natural variability and complex manufacturing process of biological medicines in general, the ‘generic’ development and approval approach is not appropriate for a biosimilar.^2,27^

Regulatory authorities such as the EMA and the United States Food and Drug Administration (FDA) have developed a regulatory approval pathway for biosimilars.^2,3^ Since the authorisation of the first biosimilar in 2006 in Europe, >40 biosimilars have received a positive opinion from the EMA and been subsequently authorised by the European Commission (EC).^5^ Since 2015, the FDA has approved over 10 biosimilars.^6^ The number of approved biosimilars will grow substantially in future years, accompanied by an increasing loss of exclusivity of biological reference products, especially in oncology.^7,8^ By providing more-affordable treatment options and introducing price competition to the market, biosimilar medicines can generate significant savings. The cumulative savings between 2016 and 2020 in the EU5 and the USA are estimated to range between 49 and 98 billion Euros.^7^ Savings derived from biosimilar market entry can relieve burdened healthcare budgets and open up budgetary room for new treatment options. Furthermore, biosimilar entry can increase patient access to biological therapies.^7,9^

Biosimilars have been integrated in cancer care for over a decade, as the first biosimilars of epoetin and filgrastim were authorised by the EMA in 2007 and 2008, respectively.^5^ The number of biosimilars available in oncology is likely to increase rapidly, with the therapeutic focus shifting from supportive care for chemotherapy to targeted, potentially life-prolonging or curative monoclonal antibodies (mAbs). The first mAb biosimilar versions in oncology, of rituximab, were approved by the EMA in 2017 (Blitzima®, Ritemvia®, Rituzena®, Truxima® by Celltrion Healthcare Hungary Kft and Rixathon®, Riximyo® by Sandoz GmbH).^5^

The mAb trastuzumab (developed by Genentech, marketed by Roche as Herceptin®) targets the human epidermal growth factor receptor 2 (HER2), and is approved for the treatment of early breast and advanced breast and gastric cancer in which HER2 is overexpressed (HER2+).^10^ HER2+ breast cancer accounts for ~15% and 20% of all breast cancers in the early and advanced stage, respectively.^11^ Trastuzumab in combination with pertuzumab and taxane chemotherapy is currently the standard first-line treatment for HER2+ metastatic breast cancer.^12^ Trastuzumab is also approved for the treatment of HER2+ early breast cancer in neoadjuvant or adjuvant settings.^11^ As the first therapeutic mAb targeted to HER2, trastuzumab has revolutionised the treatment of HER2+ breast cancer. However, its high cost (~30,500 Euros for 12 months’ treatment in an adjuvant setting and ~41,500 Euros for an average treatment period of 18.5 months in metastatic breast cancer, based on Belgian list prices for a patient that weighs 67 kg^13^) puts pressure on healthcare budgets and can restrict patient access in countries where limited or no health insurance coverage is available.^14^ Herceptin® had global sales of 6.6 billion Euros (7.5 billion USD^15^ at a 1.14 USD to 1 Euro conversion rate) in 2017 and, with the patent expiration of the intravenous reference product of Herceptin® in the European Union (EU) in 2014 and the expected patent expiration in the USA in 2019,^8^ several companies have been pursuing the development of biosimilar versions of trastuzumab. Five trastuzumab biosimilars have been approved by the EC^16–20^ and are expected to enter the European market over the course of 2018 and 2019. In the United States of America, three trastuzumab biosimilars have so far been authorised^21,22^ and are expected to enter the USA market in 2019.^8^

However, not all markets are ready to capture the potential benefits offered by biosimilars, as the uptake of biosimilars across Europe is heterogeneous and limited in some countries.^7,23^ The lack of knowledge and understanding among stakeholders about the biosimilar approval pathway and the different weight of clinical data in the development of biosimilars compared with that of an originator have been identified as hurdles for the uptake of biosimilars.^24,25^ As more biosimilars are approved and prescribed, especially in the domain of cancer with the recent approvals of therapeutic oncology biosimilars, it becomes increasingly important that healthcare providers have a good understanding about the biosimilar approval pathway and the role of clinical data in this. To address this need, the aim of this manuscript is threefold: first, to provide an overview of the biosimilar development pathway; second, to review the clinical trial parameters and published clinical data that have been collected to confirm similarity between the reference product – in this case, we will focus on trastuzumab – and its biosimilars in relation to the EMA guidelines on (mAb) biosimilar development; and, third, to provide information that can be useful in clinical decision making for prescribers and other healthcare providers who will be using trastuzumab biosimilars in clinical practice.

## The development of biosimilars

The development of biosimilar versions of previously approved biological products is based on a rigorous comparability exercise between the biosimilar and the reference product. Different from the marketing authorisation application of the reference product, the goal of the biosimilarity exercise is not to independently establish the clinical benefits of the candidate, as this has already been demonstrated for the reference product,^26^ but to demonstrate a high degree of similarity to the reference product in terms of quality characteristics, biological activity, efficacy and safety, and to exclude any clinically relevant differences that might exist between the reference product and the biosimilar.^2^

**Fig. 1:**
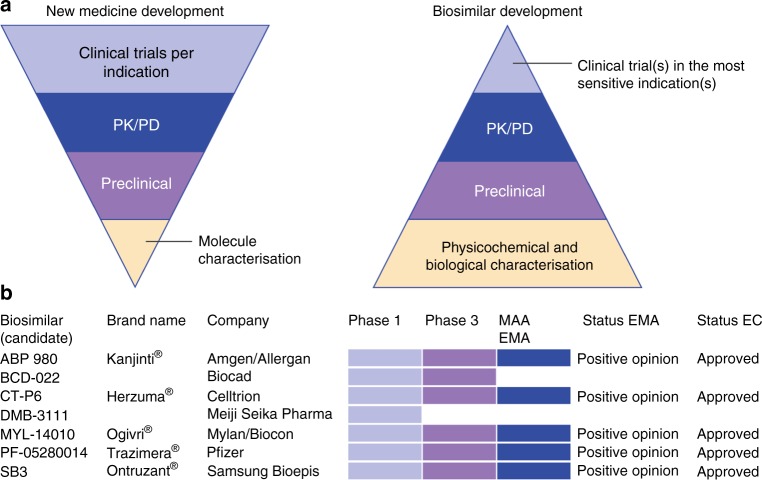
Biosimilar development: an overview of the development pathway and the different trastuzumab biosimilar(s) (candidates) approved or in clinical development. **a** New medicine versus biosimilar medicine development. Adapted from McCamish (2011) Mabs.^93^ **b** Key trastuzumab biosimilar candidates approved or in clinical development (status December 2018). EC: European Commission, EMA: European Medicines Agency, MAA: marketing authorisation application

Biosimilar development starts with a comprehensive physicochemical and biological characterisation, including a comparison of quality attributes, followed by comparative nonclinical studies.^3,4^ Further, clinical comparative testing is required to ensure similar pharmacokinetics (PK) and to confirm similar efficacy and safety to the reference product.^3^ Compared with the approval pathway for a new biological, the biosimilarity exercise places more emphasis on data from the extensive physicochemical and biological characterisation of the candidate and the comparative analytical testing with the reference product and less on those from clinical trials.^2,3,27^ The nature and extent of each step of the clinical development depends on the level of evidence obtained in the previous steps of the comparability exercise.^2,3^ The clinical package generally consists of a phase 1 study followed by at least one phase 3 study for one of the approved indications of the reference product.^3^ In some cases, confirmatory PK and pharmacodynamic (PD) studies might be sufficient to demonstrate clinical biosimilarity.^27^ At the end of the process, the biosimilar is evaluated on the overall body of evidence for biosimilarity.^3^ Figure 1 provides a schematic overview of the differences in approach between the development of a new biological and a biosimilar.

The EMA has issued several guidance documents to assist sponsors in the development of biosimilars,^28–30^ including a product-specific guideline for biosimilar mAbs.^26^ The EMA applies a case-by-case approach when guiding and evaluating the comparability exercise of a biosimilar.^26^ In this article, we discuss the clinical development of trastuzumab biosimilars in relation to EMA guidelines; some minor differences exist with FDA guidelines, but they are based on the same principle of establishing biosimilarity to the reference product.^3^ As the goal of the biosimilarity exercise is different to that of the development of a new product, the design of the clinical studies for the evaluation of biosimilars is also different to that for a new product.^3^ The studies should primarily be sensitive enough in the choice of design, population and primary endpoint such that any relevant (clinically meaningful) differences between the reference product and the biosimilar could be detected.^2,26^

## EMA biosimilar (mAb) guidelines on phase 1 PK/PD testing

The primary goal of PK studies in biosimilar development is to show comparability in PK between the biosimilar candidate and the reference product. Unless the product carries specific safety concerns, the EMA guideline on mAb biosimilar development and the EMA guideline on investigation of bioequivalence recommend performing PK testing in healthy volunteers,^26,31^ as they are less likely to show variability in PK compared with patients, and thus are a more sensitive and homogenous group in which to detect potential clinically meaningful differences in PK characteristics between the two products.^26^ It is also advisable to collect supportive PK data in the clinical patient studies. A single-dose study with a parallel group design is advised, owing to the long half-life of mAbs and the potential impact of immunogenicity. In addition to conventional PK parameters, including the area under the curve (AUC) and *C*_max_, it is advisable to measure safety and immunogenicity parameters in parallel, such as the presence of antidrug antibodies.^26^

PK studies can, when available, be combined with PD endpoints, which can add valuable information for the comparability exercise, especially if the PD endpoints are sensitive enough to detect small differences between the biosimilar and the reference product, and if they can be measured with sufficient precision.^26^ PD testing can potentially also be considered as pivotal evidence to establish clinical biosimilarity, provided that a clear dose–response relationship can be shown and a PD marker that is accepted as surrogate marker of a patient outcome is available.^26^ If this is not the case, similar clinical efficacy needs to be demonstrated in a phase 3 comparative trial.^26^

## EMA biosimilar (mAb) guidelines on phase 3 studies

The primary objective of a phase 3 biosimilarity trial is to demonstrate similar clinical efficacy and safety between the candidate and its reference product. To this end, the EMA advises conducting an adequately powered, randomised, parallel group comparative clinical trial, preferably double-blind, with an equivalence study design, for at least one representative indication.^26^

To allow detection of potential differences between the candidate biosimilar and the reference product, the EMA advises conducting the phase 3 trial in the most sensitive and homogenous patient population.^26^ Reducing patient-related factors and disease-related factors (e.g., differences in disease severity or different previous lines of treatment) to a minimum will allow potential differences to be attributed to the product, rather than to the patient or the disease.^26^

Progression-free survival (PFS) and overall survival (OS) are conventional endpoints that are used to demonstrate efficacy in cancer indications. However, it might not be feasible to use these as primary endpoints for phase 3 biosimilarity trials, as they require a long follow-up period. Furthermore, they might not be sensitive enough to demonstrate comparability, as they can be influenced by non-product-related factors, such as tumour burden, performance status and previous and or later lines of treatment. Therefore, the use of a sensitive endpoint that measures shorter-term activity is recommended, although, when feasible, it is advisable to record PFS and OS in addition.^26^

As well as comparable efficacy, comparable safety needs to be demonstrated during phase 3 evaluation. Adverse events, particularly those described for the reference product, and immunogenicity, by measuring antidrug antibodies, should be assessed.^26^

## Trastuzumab biosimilars in clinical development

Several trastuzumab biosimilar candidates have been developed, with at least seven of them entering clinical development (Fig. 1). Five developers, Samsung Bioepis (SB3), Celltrion (CT-P6), Mylan/Biocon (MYL-1401O), Amgen/Allergan (ABP 980) and Pfizer (PF-05280014) have submitted their candidate for marketing authorisation to the EMA. In September 2017, the committee for medicinal products for human use (CHMP) recommended the granting of a marketing authorisation for Samsung Bioepis’ candidate, SB3 (Ontruzant®).^32^ Four other recommendations for approval followed for Celltrion’s product (CT-P6, Herzuma®), ABP 980 from Amgen/Allergan (Kanjinti®), Pfizer’s candidate (PF-05280014, Trazimera®) and Mylan’s product (MYL-1401O, Ogivri®).^33–36^ These products received a marketing authorisation from the EC^16–20^ and are gradually entering the European market.

Mylan/Biocon, Celltrion, Amgen/Allergan, Samsung Bioepis and Pfizer also submitted a Biologics License Application (BLA) for their candidate to the FDA.^15,37^ In December 2017, the FDA announced the approval of Ogivri® (MYL-1401O) as first trastuzumab biosimilar in the USA.^21^ Herzuma® (CT-P6), Ontruzant® (SB3) and Trazimera® (PF-05280014) have been approved in December 2018, January 2019 and March 2019, respectively.^22^

Some of these recently EC/FDA-approved trastuzumab biosimilars or candidates are already on the market in other regions of the world. For example, the candidate co-developed by Mylan and Biocon was launched in India in 2013 (under the brand names Hertraz® and CANMab®, respectively). Celltrion has marketed its candidate as Herzuma® in South Korea since 2014 and Biocad’s product has been marketed in Russia under the brand name HERtiCAD® since 2016.^15^ As the regulatory approval process is less stringent in countries such as Russia and India, these products should not be considered as biosimilars before being assessed by regulatory authorities such as the EMA and FDA.^15^

## Clinical data from phase 1 trastuzumab biosimilar trials

All seven trastuzumab biosimilar candidates showed an equivalent PK profile to the reference product, as primary PK outcomes fell within the pre-specified bioequivalence margin of 80–125%, with a 90% Confidence Interval (CI). Although EMA guidelines recommend PK testing for mAbs in healthy volunteers, Celltrion and Biocad performed PK testing in HER2+ patients with metastatic breast cancer.^38,39^ Other developers, however, followed the EMA guidelines and conducted PK testing for their candidate in healthy volunteers.^40–45^ Table 2 provides an overview of the trial parameters and phase 1 PK outcomes for the different biosimilar candidates. The patient population size varied from 46 (BCD-022) to 174 (CT-P6) healthy volunteers or patients.

**Table 2:** Phase 1 PK equivalence results for the trastuzumab biosimilar(s) (candidates)

Biosimilar (candidate)	Study population	Comparator	Dosing	Primary endpoints	Bioequivalence margins	Primary outcome results	Equivalence to RP established?	Ref.
ABP 980 (Amgen/Allergan)	HV (*n* = 157)	EU-RP+US-RP	1 × 6 mg/kg	AUC_inf_ *C*_max_	90% CI, 80–125%	1.00 (0.95, 1.06) 1.06 (0.99, 1.12) 0.99 (0.95, 1.03) 1.04 (0.99, 1.08)	Equivalent to EU-RP and US-RP	^40,41^
BCD-022^+^ (Biocad)	HER2+MBC (*n* = 46)	RP	1 × 8 mg/kg	AUC_0–504_	90% CI, 80–125%	80.42-120.87%	Equivalent to RP	^38^
CT-P6 (Celltrion)	HER2+MBC (*n* = 174)	RP	1 × 8 mg/kg, 8 × 6 mg/kg	AUC_SS_ at cycle 8	90% CI, 80–125%	104.57 (93.64, 116.78)	Equivalent to RP	^39^
DMB-3111 (Meiji Seika)	HV (*n* = 70)	RP	1 × 6 mg/kg	*C*_max_ AUC_inf_ t_1/2_	90% CI, 80–125%	log(0.9384)-log(1.0554) log(0.9429)-log(1.0627) log(0.9450)-log(1.0777)	Equivalent to RP	^42^
MYL-1401O (Mylan/Biocon)	HV (*n* = 120)	EU-RP+US-RP	1 × 8 mg/kg	AUC_0–inf_ AUC_0–last_ *C*_max_	90% CI, 80–125%	0.97 (91.17, 102.97) 0.96 (89.96, 101.94) 0.97 (91.31, 103.05) 0.96 (90.34, 102.29) 1.04 (99.00, 109.82) 1.02 (96.42, 107.26)	Equivalent to EU-RP and US-RP	^43^
PF-05280014 (Pfizer)	HV (*n* = 105)	EU-RP+US-RP	1 × 6 mg/kg	AUC_0–last_ AUC_0–inf_ *C*_max_	90% CI, 80–125%	92.66 (86.44, 99.34) 99.94 (93.08, 107.31) 92.15 (86.03, 98.69) 99.83 (93.06, 107.09) 91.49 (85.32, 98.09) 97.41 (90.71, 104.62)	Equivalent to EU-RP and US-RP	^44^
SB3 (Samsung Bioepis)	HV (*n* = 109)	EU-RP+US-RP	1 × 6 mg/kg	AUC_0–inf_ AUC_0–last_ *C*_max_	90% CI, 80–125%	0.969 (0.908, 1.034) 0.930 (0.872, 0.991) 0.971 (0.911, 1.034) 0.934 (0.878, 0.994) 1.001 (0.935, 1.072) 0.988 (0.924, 1.057)	Equivalent to EU-RP and US-RP	^45^

*AUC* area under the curve; *CI* confidence interval; *EBC* early breast cancer; *HV* healthy volunteers; *MBC* metastatic breast cancer; *n* number; *RP* reference product

^+^BCD-022 is authorised in Russia, but has not been submitted to FDA or EMA and most likely would not be considered as a biosimilar following stringent FDA or EMA requirements

Data are derived from published scientific literature (full text or abstract)

The reported safety results were overall comparable between the respective biosimilar and the trastuzumab reference product. An overview of phase 1 safety outcomes is shown in Table 3. Amgen/Allergan reported a treatment-emergent adverse event (TEAE) incidence of 84%, 75%, and 78% in subjects receiving their candidate (ABP 980), USA-sourced trastuzumab and EU-sourced trastuzumab, respectively.^40,41^ PF-05280014, Pfizer’s candidate, showed a numerically higher incidence of pyrexia in the biosimilar treatment arm, but the severity of this adverse event was reported to be generally mild.^44^ Phase 1 comparative testing of SB3 showed a numerical higher TEAE incidence for the EU-sourced trastuzumab and the USA-sourced trastuzumab compared to SB3 (44.4%, 61.1%, and 36.1%, respectively).^45^ Events related to cardiac function – patients treated with trastuzumab have a small to moderately increased risk of cardiotoxicity – were reported for some of the candidates. In addition, a phase 1 study for the candidate of Amgen/Allergan (at that time referred to as FTMB, developed by Synthon^46^) by Wisman et al. investigated the cardiotoxicity of ABP 980 in healthy volunteers and added a dose-escalation part while monitoring the cardiac function.^47^ During the dose-escalation period, no safety concerns that would impede progression of the study towards its bioequivalence phase were detected using either the biosimilar or the reference product.

**Table 3:** Phase I safety results for the trastuzumab biosimilar(s) (candidates)

Biosimilar (candidate)	Adverse events	Cardiotoxicity	Antidrug antibody formation	Source/Ref
ABP 980 (Amgen/Allergan)	TEAEs occurred in 84%, 75% and 78% of subjects receiving ABP 980, US-RP and EU-RP, respectively. One grade 3 SAE in EU-RP group.	NR	No ADA were detected	Abstract^40,41^
(FTMB)*	No differences in AEs between groups (double-blinded, dose-escalation part). In the open-label part, flu-like symptoms and fatigue more frequently reported for the biosimilar.	No signs of cardiotoxicity	No ADA were detected	Full text^47^
BCD-022^+^ (Biocad)	No significant differences between groups.	NR	NR	Abstract^38^
CT-P6 (Celltrion)	SAEs in 15.8% and 20.9% in CT-P6 and RP group, respectively. TEAEs in 40,8% for CT-P6 and 46.3%, for RP group.	2.6% cardiotoxicity in CT-P6 group, 7.5% in RP group	NR	Abstract^39^
DMB-3111 (Meiji Seika)	No significant differences between groups.	NR	No subjects developed ADA	Full text^42^
MYL-1401O (Mylan/Biocon)	31, 28, 24 subjects experienced in total 227 (91, 80, 56) TEAEs, (mild to moderate in severity) in the biosimilar, EU-RP and US-RP group, respectively. No serious AEs detected. No significant differences between groups.	NR	No subjects developed ADA	Abstract^43^
PF-05280014 (Pfizer)	Numerically higher incidence of pyrexia in biosimilar arm, but severity generally mild. (in 10, 3, 2 patients in biosimilar, EU-RP, US-RP, respectively)	No unusual LVEF values reported	One case of ADA after EU-RP	Full text^44^
SB3 (Samsung Bioepis)	AEs: 69.4%, 63.9%, 69.4%** TEAEs: 36.1%, 44.4%, and 61.1%** Infusion related reactions: 9, 8, 16**	NR	No subjects tested positive for ADA	Full text^45^

*ADA* antidrug antibodies; *AEs* adverse events; *LVEF* left ventricular ejection fraction; *NR* not reported; *RP* reference product; *SAE* serious adverse event; *TEAE* treatment emergent serious adverse event

^*^FTMB: biosimilar candidate developed by Synthon Biopharmaceuticals. Synthon entered into a global license agreement with Amgen/Watson in 2012. Amgen/Watson continued further development (incl. phase 3 clinical trial), global manufacturing and commercialisation^46^

**In SB3, EU RP and US RP group, respectively

^+^BCD-022 is authorised in Russia, but has not been submitted to FDA or EMA and most likely would not be considered as a biosimilar following stringent FDA or EMA requirements

Data are derived from published scientific literature (full text or abstract)

A lack of clinically validated PD markers for trastuzumab makes it necessary to confirm clinical comparability via a phase 3 trial.^26,48^

## Phase 3 efficacy and safety testing for trastuzumab biosimilar candidates

Six trastuzumab biosimilar candidates have been tested in phase 3 trials. Reported phase 3 data are in support of biosimilarity between the candidates and the trastuzumab reference product. For five candidates equivalence in efficacy to trastuzumab was considered to be established (for ABP 980, CT-P6, MYL-1401O, PF-05280014 and SB3).^49–56^ For BCD-022, non-inferiority in efficacy to trastuzumab was demonstrated in metastatic breast cancer patients.^57^ Differences in the selected patient population, primary endpoints and trial design exist between the different candidates. Table 4 shows the trial parameters and a summary of comparative efficacy results for the phase 3 trials. Candidate-specific phase 3 results are further discussed in the supplementary information of this article. The reported safety data of phase 3 testing can be viewed in Table 5.

**Table 4:** Phase 3 trial parameters and primary endpoint results for the trastuzumab biosimilar(s) (candidates)

Biosimilar (candidate)	Company	*n* patients	Patient setting	Primary endpoint	Equivalence (E)/ Non-inferiority (NI) margin	Primary endpoint results	Ref.	EU MAA/MA Status^15^
ABP 980	Amgen/ Allergan	725	Neoadjuvant+adjuvant EBC	tpCR	E margin: −13%, +13% with 90% CI for RD^°^; 0.759, 1.318 with 90% CI for RR^°°^	RD: 7.3% (1.2, 13.4)^*^ 5.8% (−0.5, 12.0)^**^ RR: 1.19 (1.033, 1.366)^*^ 1.14 (0.993, 1.312)^**^	^55,56^	Approved as Kanjinti® on 16/05/2018^19^
BCD-022^+^	Biocad	126	MBC	ORR	NI margin: −20% with 95% CI for RD in ORR	RD: −0.13% (−19.83%, 18.35%)	^57^	No application
CT-P6^x^	Celltrion	475	MBC	ORR	E margin: −0.15, 0.15 with 95% CI for RD^°^	RD: 5% (−0.14, 0.04)	^66^	Approved as Herzuma® on 08/02/2018^18^
		549	Neoadjuvant + adjuvant EBC	tpCR	E margin: −0.15, 0.15 with 95% CI for RD^°^ 0.74, 1.35 with 95% CI for RR^°°^	RD: −0.04 (−0.12, 0.05) RR: 0.93 (0.78, 1.11)	^67^	
MYL-1401O	Mylan/ Biocon	500	MBC	ORR	E margin: −15%, +15% with 95% CI for RD^°^ 0.81, 1.24 with 90% CI for RR^°°^	RD: 5.53 (−3.08, 14.04) RR: 1.09 (0.974, 1.211)	^61,62^	Approved as Ogivri® on 12/12/2018^20^
PF-05280014^~^	Pfizer	707	MBC	ORR	E margin: 0.8, 1.25 with 95% CI for RR^°°^	RR: 0.940 (0.842, 1.049)	^65^	Approved as Trazimera® on 26/07/2018^17^
		226	Neoadjuvant EBC	% pts with cycle 5 *C*_trough_ >20 μg/mL	NI margin: −12.5% with 95% CI for stratified difference in *C*_trough_	92.1% for PF-05280014 vs 93.3% for RP-EU (−8.02%, 6.49%)	^64^	
SB3	Samsung Bioepis	800	Neoadjuvant + adjuvant EBC	bpCR	E margin: −13%, +13% with 95% CI for RD^°^; 0.785, 1.546 with 95% CI for RR^°°^	RD: 10,70% (4.13, 17.26) RR: 1.259 (1.085, 1.460)	^54,63^	Approved as Ontruzant® on 15/11/2017^16^

*bpCR* breast pathological complete response; *CI* confidence interval; *E* equivalence; *EBC* early breast cancer; *MA* marketing authorisation; *MAA* marketing authorisation application; *MBC* metastatic breast cancer; *n* number; *NI* non-inferiority; *NR* not reported; *ORR* overall response rate; *RD* risk difference; *RP* reference product; *RR* risk ratio; *tpCR* total pathological complete response (breast + lymph nodes)

Data are derived from published scientific literature (full text or abstract)

^*^Based on local review

^**^Based on central independent review

^°^EMA advised

^°°^FDA advised

^+^BCD-022 is authorised in Russia, but has not been submitted to FDA or EMA and most likely would not be considered as a biosimilar following stringent FDA or EMA requirements

^x^The phase 3 data in MBC for CT-P6 were not submitted to EMA as part of the marketing authorisation application and were thus not evaluated when assessing the totality of evidence for biosimilarity^50^

^~^The pivotal phase 3 trial for PF-05280014 was conducted in the MBC setting. Supportive efficacy data have been gathered in a phase 3 clinical trial in patients with early breast cancer in the neoadjuvant setting (PK endpoint as primary endpoint^52^)

**Table 5:** Phase 3 safety results for the trastuzumab biosimilar(s) (candidates)

Biosimilar (candidate)	Adverse events	Cardiotoxicity	Antidrug antibody detection	Ref.
ABP 980 (Amgen/Allergan)	≥ 1 AE: 80.2% vs 79.5%, Grade ≥ 3 AE: 14.8% vs 14.1% for ABP 980 and RP, respectively°	Six patients in the ABP 980 group and one in the RP group had cardiac failure adverse events. All events were grade 1 or 2, and patients completed planned doses with no worsening of the cardiac failure event°	Two patients in each group developed binding antibodies. Neither tested positive for neutralising antibodies°	^55,56^
	AE: 52.0% vs 57.3% for RP-RP group and switch group, Grade ≥ 3 AE: 10 in each group°°	One patient (0.6%) with cardiac failure in each group°°	One patient with binding, non-neutralising ADA (switch group)°°	^82^
BCD-02^+^ (Biocad)	No statistically significant difference in AEs, including SAEs, between groups	Tachycardia (34.92% vs 19.67%), arterial hypertension (20.63 vs 18.03%) atrial fibrillation (0% vs 3.28%), extrasystoles (0% vs 1.64%), aggravated myocardiodystrophy (1.59% vs 0%)	Neutralising ADA in one patient in each group	^57^
CT-P6^x^ (Celltrion)	AEs comparable between groups*	Cardiotoxicity in 8 (3.3%) and 10 (4.3%) patients in biosimilar and RP group, respectively*	NR*	^66^
	STEAE: 7% vs 8% for CT-P6 and RP group Grade ≥ 3 TEAE: 6% vs 8% for CT-P6 and RP group**	TEAEs owing to heart failure in 2% vs 1% for CT-P6 and RP group, respectively. Of these, one patient (RP group) withdrawn from study (confirmed decrease in LVEF). One grade 1 heart failure (CT-P6 group), but no substantial decrease in LVEF**	All post infusion ADA tests were negative**	^67^
MYL-1401O (Mylan/Biocon)	TEAEs and SAEs similar between groups	No difference in median LVEF between groups	ADA similar between groups	^61,62^
PF-05280014 (Pfizer)^~^	SAEs similar in both arms*	NR*	One patient developed ADA (EU-RP)*	^65^
	Grade 3–4 TEAEs: 38.1% vs 45.5% for PF-05280014 and RP**	No TEAEs of congestive heart failure or clinically significant LVEF abnormalities were reported in either arm. No notable differences between the treatment groups in mean LVEF results.**	No patients with ADA for PF-05280014 vs one patient for RP**	^64^
SB3 (Samsung Bioepis)	SAEs: 10.5% vs 10.7% for SB3 and RP**	Two patients in SB3 group presented with CHF**	ADA 0.7% vs 0.0% for SB3 and RP**	^54^
	TEAEs (97.5% vs 96.1% for SB3 and RP) similar between groups***	14 LVSD events in 11 (2.5%) patients in biosimilar group, 9 LVSD events in 8 (1.8%) patients in RP group. Four patients (three in SB3, one in RP) reported CHF***	0.7% in both groups***	^63^

*ADA* antidrug antibodies; *AE* adverse event; *CHF* congestive heart failure; *LVEF* left ventricular ejection fraction; *LVSD* asymptomatic left ventricular systolic dysfunction; *NR* not reported; *RP* reference product; *SAE* serious adverse event; *TEAE* treatment emergent serious adverse event

Data are derived from published scientific literature (full text or abstract)

^°^Results from neoadjuvant setting

^°°^Results from the single switch treatment arm vs continuing arm in adjuvant phase of the study

^*^Reported results are safety results of the phase 3 trial in metastatic breast cancer population

^**^Reported results are safety results of the phase 3 trial in early breast cancer patients (neoadjuvant period)

^***^Reported results are safety results of the phase 3 trial in early breast cancer patients (neoadjuvant + adjuvant period)

^+^BCD-022 is authorised in Russia, but has not been submitted to FDA or EMA and most likely would not be considered as a biosimilar following stringent FDA or EMA requirements

^x^The phase 3 data in MBC for CT-P6 were not submitted to EMA as part of the marketing authorisation application and were thus not evaluated when assessing the totality of evidence for biosimilarity^50^

^~^The pivotal phase 3 trial for PF-05280014 was conducted in the MBC setting. Supportive efficacy data have been gathered in a phase 3 clinical trial in patients with early breast cancer in the neoadjuvant setting (PK endpoint as primary endpoint)^52^

A first point of variation in the phase 3 clinical development of the different trastuzumab biosimilar candidates is the selected patient population. As trastuzumab is approved in the treatment of patients with metastatic breast cancer, early breast cancer and metastatic gastric cancer, the sponsor can decide between different patient settings in which to test its candidate. Without specifying its preference for metastatic breast cancer or early breast cancer, the EMA advises conducting phase 3 testing in the most sensitive and homogeneous population.^26^ It could be argued that patients with metastatic breast cancer potentially represent a less homogeneous, and thus less sensitive, group owing to a number of confounding factors, such as location of metastases, comorbidities, disease severity and the number and type of prior therapies.^48,58–60^ Unless adequately controlled for in the statistical design of the study, this heterogeneity is likely to have an impact on the validity of the trial’s conclusions.^48^ In this regard, early breast cancer might represent a more sensitive and homogeneous population, as patients with early breast cancer generally have fewer confounding characteristics (little or no prior therapy and generally a better performance status).^48,58–60^ Mylan/Biocon and Biocad chose to conduct their phase 3 trial in patients with metastatic breast cancer,^57,61,62^ whereas Samsung Bioepis and Amgen/Allergan performed their phase 3 trial in early breast cancer patients.^54–56,63^ Pfizer and Celltrion conducted two phase 3 trials, one for each patient setting.^64–67^ The phase 3 Pfizer trial in early breast cancer was based on a PK primary endpoint.^64^ Celltrion’s phase 3 trial in metastatic breast cancer was not submitted to EMA as part of the marketing authorisation application. Table 4 provides an overview of phase 3 trial parameters for the different candidates. The patient population size varied from 126 (BCD-022) to 800 (SB3) patients.

A second point of variation in clinical testing is the choice of clinical trial endpoint. According to the product-specific EMA guideline of biosimilar mAbs, the clinical endpoint that is most sensitive at detecting product-related differences should be selected.^26^ A surrogate clinical endpoint that measures shorter-term activity as the primary endpoint may be considered.^26^ Response rates such as overall response rate (ORR; the proportion of patients in whom a complete response (CR) or partial response (PR) was observed) and pathological complete response (pCR) might be suitable for detecting meaningful differences in activity between the candidate and its reference product, if any.^26^ In the case of trastuzumab biosimilars, pCR could be deemed as the more favourable endpoint, as it has been shown to correlate with long-term survival in patients with early breast cancer.^59,68^ A pooled analysis of 12 randomised controlled trials of neoadjuvant therapy in early breast cancer with ~12,000 patients showed that pCR was associated with a long-term survival outcome.^69^ In this regard, pCR in early breast cancer (Amgen/Allergan, Celltrion, Samsung Bioepis) might be a more desirable approach in establishing clinical biosimilarity than ORR in metastatic breast cancer (Biocon, Mylan, Pfizer).

The definition of the primary endpoint also differs across studies. Of the three sponsors who chose to conduct their (main) phase 3 trial in early breast cancer, two – Amgen/Allergan and Celltrion – selected pCR in both breast tissue and axillary lymph nodes (total pCR (tpCR));^55,56,67^ the third, Samsung Bioepis, chose pCR in breast tissue alone (breast pCR (bpCR)) as the primary endpoint.^54^ The tpCR could potentially be deemed as a more convincing primary endpoint by the prescriber, as the eradication of tumour from both breast and lymph nodes has been shown to have a stronger association with improved long-term survival outcomes, than eradication from the breast alone.^69,70^

The selected endpoints for the evaluation of biosimilarity might be less acceptable for oncologists, as they are different from the conventional efficacy endpoints that show patient benefit. However, the goal of the comparability exercise is to demonstrate biosimilarity rather than patient benefit, which has already been demonstrated for the reference product. Therefore, it is important to inform clinicians and other healthcare providers about the rationale behind the biosimilar development pathway and its stepwise approach.

The choice between an equivalence or a non-inferiority trial design is a third point of variation. As the biosimilar concept is based on demonstrating similarity of the biosimilar to its reference product, the EMA advises an equivalence study design for phase 3 testing of mAb biosimilars.^26^ An equivalence trial is intended to demonstrate that neither the candidate nor the comparator (the reference product) is inferior or superior to the other, by showing that any difference in response between the two is likely to lie within a pre-specified range of clinically acceptable differences.^71^ Most of the companies have adhered to EMA guidance by deciding on a two-sided equivalence test to demonstrate similar clinical efficacy and safety to trastuzumab.

In contrast, Biocad’s candidate (BCD-022) was tested in a non-inferiority trial.^57^ A non-inferiority trial tends to require a smaller sample size than equivalence testing, but only rules out inferiority, not potential superiority, to the reference product.^71^ The clinical trial of BCD-022 was performed in a relatively small patient cohort of 126 patients with metastatic breast cancer with the non-inferiority margin set at −20% with a 95% CI for risk difference in ORR. The results showed that the lower limit of the 95% CI for risk difference in ORR between the groups (−19.83%) did not exceed the non-inferiority margin, demonstrating non-inferiority to trastuzumab.^57^ BCD-022 was approved by the Ministry of Health of the Russian Federation at the beginning of 2016, but has not been submitted for approval in Europe or in the USA.^15^ Based on the results of this study, it is unlikely that BCD-022 would be granted marketing authorisation as a biosimilar by rigorous EMA standards. Pfizer also performed a non-inferiority phase 3 trial (in a neoadjuvant setting, *C*_trough_ at steady state as the primary endpoint with secondary efficacy endpoints).^64^ However, Pfizer’s pivotal phase 3 trial adhered to an equivalence design (in patients with metastatic breast cancer, with ORR as the primary endpoint).^65^

For SB3, the lower boundary of the 95% CI for risk difference in bpCR (95% CI: 4.13, 17.26) fell within the predefined equivalence margin (−13%, +13%), while the upper boundary exceeded the equivalence margin,^54^ ruling out non-inferiority but not potential superiority. The boundaries of the 95% CI for the ratio of bpCR (95% CI: 1.085, 1.460) fell within the predefined equivalence margin (0.785, 1.546), demonstrating equivalence.^54^ For ABP 980, based on predefined local review, the lower boundaries of the 90% CI for both risk difference and risk ratio of pCR fell within the pre-specified equivalence margins and the upper boundaries of the CI for both exceeded the equivalence margins, thereby excluding non-inferiority but not potential superiority.^55,56^ In sensitivity analyses based on central independent review of tumour samples by blinded pathologists, the risk difference and risk ratio of pCR fell within the equivalence margins.^55,56^ These observations for SB3 and ABP 980 were deemed at least partially confounded by a small downward shift in ADCC activity in the EU trastuzumab reference product batches (as described in the literature^72^) that were used in their phase 3 comparative trial, as stated in the European public assessment report of both Ontruzant® (SB3) and Kajinti® (ABP 980).^49,53^ Both SB3 and ABP 980 have been approved as a biosimilar of trastuzumab, as the overall body of evidence sufficiently demonstrated biosimilarity compared to the reference product.^49,53^

## Extrapolation of indications

A biosimilar candidate can be considered for approval for one or more indications for which the reference product is approved, without itself being subjected to clinical testing for all of these indications. This regulatory concept is called extrapolation of indications.^26,73^ The main rationale for extrapolation of data to other indications is to avoid unnecessary clinical studies.^74,75^ Extrapolation is decided on a case-by-case basis, taking into account the overall evidence gathered in the comparability exercise of the candidate, including safety, efficacy and immunogenicity data, in a key indication that is suitable to detect clinically meaningful differences, and the scientific justification for extrapolating.^26^ The scientific justification requires detailed knowledge of the mechanism of action and the targets involved, the PK profile, immunogenicity and adverse events that might be expected in the different indications.^26,28,73^ If the mechanism of action is complex and involves multiple receptors or binding sites that contribute differently to the different therapeutic indications, additional data might be required to allow for extrapolation.^75^

Extrapolation is an established regulatory principle that is not only applied in the context of biosimilars, but also for example when a new formulation of a licensed product is developed.^73,74^ For instance, Roche has developed a subcutaneous formulation of trastuzumab, which was clinically tested in the neoadjuvant setting and was approved in Europe in 2013 for all indications after extrapolating to the metastatic setting.^73,76^ Although the concept of extrapolation is essential in the biosimilar development pathway, the use of extrapolation of indication has raised concerns among healthcare providers.^24,73^ In particular, if the reference product is used across different therapeutic areas (e.g., autoimmune disease and oncology), different pathologies (e.g., breast cancer and gastric cancer) or different disease settings (e.g., first-line and second-line), extrapolation can be perceived as challenging. The first biosimilar of rituximab, Truxima®, was approved for all indications of rituximab, including indications in oncology, after it was tested in a pivotal phase 3 trial in rheumatoid arthritis patients, and supportive data were gathered in patients with advanced follicular lymphoma (similarity in PK and non-inferiority in efficacy).^77^ For trastuzumab biosimilars, extrapolation has already been granted by the EMA both from early breast cancer to metastatic breast cancer and metastatic gastric cancer (SB3, ABP 980 and CT-P6) as well as from metastatic breast cancer to early breast cancer and metastatic gastric cancer (MYL-1401O), based on the totality of evidence for biosimilarity.^49–51,53^

## Clinical implementation and strategic considerations of trastuzumab biosimilars

### Switching between the reference product and biosimilar versions of trastuzumab

Initiating treatment with an approved trastuzumab biosimilar is as safe and effective as initiating treatment with the reference product. However, questions have been raised about switching between a reference product and its biosimilar or between biosimilars of the same reference product.^78^ Although no issues have been identified thus far with switching from a reference product to its biosimilar,^79^ a concern is that switching could potentially lead to increased immunogenicity, owing to the subsequent exposure to potentially different sets of epitopes owing to minor differences that might exist between the reference product and the biosimilar. An increasing amount of data from both phase 3 extension trials and real-world studies evaluating the impact of switching are available for biosimilars of various products, including infliximab, etanercept and adalimumab.^79,80^

In 2016, the European Society for Medical Oncology published a position paper about biosimilars, indicating that the decision to switch from the reference product to a biosimilar should be taken by the physician.^81^ Furthermore, when switching, the patient should be adequately informed and subsequently monitored, allowing any adverse events to be traced to the relevant product.^81^

Thus far, eight switching studies with anticancer mAb biosimilars have been published.^80^ Seven of these studies were conducted for rituximab biosimilars and one study has been conducted for a trastuzumab biosimilar, ABP 980.^80^ Reported results indicated that switching from the trastuzumab reference product to ABP 980 following surgery was safe in patients with early breast cancer (single switch, parallel arm, *n* = 171 in each arm). The frequency and severity of adverse events did not increase, no unexpected safety signals were noted and no increased incidence of antidrug antibodies was reported.^82^

Trastuzumab is a relatively safe molecule with a low immunogenic potential for a mAb, limiting the risk of immunogenicity-related adverse events. Although switching will normally occur less frequently than for diseases requiring lifelong chronic biological treatment, it still remains a possibility in practice, as trastuzumab is administered for up to 1 year in early breast cancer or until disease progression in metastatic breast cancer and metastatic gastric cancer.^10^ Although no safety issues are to be expected when switching, a cost/benefit assessment could be of interest to investigate the trade-off between the savings from switching to a less expensive version and the costs from implementing the switch, given the relatively short treatment period.

### Strategic considerations

The different companies developing trastuzumab biosimilars have followed a variety of clinical development pathways, demonstrating the leeway given to biosimilar sponsors in determining the clinical development strategy. There might be various reasons for these different approaches, although we believe that there are also important strategic considerations behind the decisions. These considerations could apply to obtaining marketing authorisation as quickly as possible or supporting the biosimilar in such a way that it will receive higher product acceptance by stakeholders and more support in the market. Running a trastuzumab biosimilarity trial for metastatic breast cancer might benefit from faster patient accrual and possibly more-quickly attainable clinically relevant endpoints compared with early breast cancer, for example. Once licensed, early breast cancer will be an extrapolated indication for these biosimilars (if decided so by the EMA), but with potentially more reluctance among prescribers to accept this. On the other hand, running a trial for early breast cancer might be more difficult in terms of attracting patients, but clear proof in this indication might be more convincing and avoid discussions by healthcare providers relating to extrapolated indications once the product is on the market.

### Potential implications of the market entry of trastuzumab biosimilars

Roche has developed a subcutaneous formulation of trastuzumab, which is reported to be more time efficient (shorter patient chair time and active healthcare professional time) than intravenous infusion.^83^ When the total treatment costs of intravenous trastuzumab and the subcutaneous version were compared in the Netherlands in 2017, the subcutaneous preparation and administration cost (including staff, material, premedication and societal costs) was found to be 45% lower than the intravenous administration. However, this cost accounts for a limited share (<10%) of the total treatment cost (preparation and administration cost plus the medicine price).^84^ The administration cost is thus unlikely to outweigh the potential difference in medicine prices (lower priced intravenous reference product due to competition or lower priced intravenous biosimilar, versus patent protected, more-expensive subcutaneous version).

The arrival of biosimilars can potentially encourage manufacturers to invest in the development of new, innovative products.^7,85^ Besides the subcutaneous formulation, Roche has developed additional anti-HER2+ biopharmaceuticals, Perjeta® and Kadcyla®.^85,86^ Perjeta® blocks receptor dimerisation by targeting domain II of the extracellular component of HER2, whereas Kadcyla® combines the actions of trastuzumab with an anti-microtubule cytotoxic agent to facilitate intracellular delivery of the drug.^86,87^ Both therapies are implemented in clinical practice and are even more expensive than Herceptin®, with treatment costs of ~75,000 Euros (18.5 months of treatment with Perjeta®) and 57,000 euros (10 months of treatment with Kadcyla®), based on Belgian list prices.^13^ Despite these innovations, trastuzumab is likely to remain a cornerstone in the treatment of HER2+ cancer^86,88^ and trastuzumab biosimilars can have a significant role in cost containment. Biosimilars have a good value proposition, as their adoption allows to reduce the healthcare budgetary burden and or potentially relocate funds to new therapies.^89^ Biosimilar discounts can be as high as 60–90% of the originator list price (depending on the product class and country).^90^ Furthermore, the increased competition can drive down prices not only for the reference product, but also for the total therapy area segment, as previously identified by IMS Health for other biosimilar classes.^9,91^

Beyond financial benefit, the use of biosimilars ultimately provides patient benefit, too. Biosimilar market entry has previously been shown to improve patient access to biological medicines (an increase in the number of treated patients and/or more timely access to therapy).^7^ For example, in Sweden, the launch of the biosimilar filgrastim led to the reassessment of physician guidance on granulocyte colony-stimulating factor prescribing, and promoted filgrastim to first-line supportive care in cancer. Subsequently, the uptake of filgrastim increased fivefold.^7^ As trastuzumab is not currently widely accessible around the world owing to its high cost,^14^ the entry of more-affordable versions of trastuzumab could open up treatment access. Accordingly, this requires a sufficiently reduced price of the trastuzumab biosimilars and/or the reference product itself.^92^ In a physician survey in the USA and emerging markets by Lammers and colleagues in 2014, nearly half of the oncologists questioned reported that they would increase the use of HER2 targeted therapy across treatment settings if a trastuzumab biosimilar was available at a lower cost.^14^ The extent of the savings that can be realised and the improvement in patient access to trastuzumab will ultimately depend on the understanding and subsequent confidence of oncologists to prescribe trastuzumab biosimilars. Physicians may expect products that are equally safe, qualitative and effective as the reference product, and that have been rigorously evaluated by regulatory authorities such as the EMA, based on sound scientific principles.

The different routes taken in the clinical development of trastuzumab biosimilars demonstrate that sponsors have some flexibility in setting up the clinical development of their product. This should, however, not influence the confidence in a trastuzumab biosimilar once approved. Although a hierarchy could be made based on the clinical assessment of biosimilars,^60^ this would not automatically allow the ranking of one trastuzumab biosimilar above another, as biosimilarity is first established through analytical studies and further evaluated on the total body of evidence, not solely on the design and results of the clinical studies. Furthermore, this would not correspond with the concept of biosimilarity. One biosimilar might have a more extensive or sensitive clinical data package than another, but this does not mean that this biosimilar should be considered more similar to the reference product than the other, as all candidates need to prove their overall similarity to the reference product. However, a more elaborate and sensitive clinical package might gain acceptance more convincingly by healthcare providers.

## Conclusions

Several trastuzumab biosimilars are gradually entering the European market. These biosimilars represent an important opportunity for society in terms of cost savings and for patients by opening up treatment access. Although some differences do exist between the clinical development packages (in terms of trial setting, clinical endpoint and patient population) of the trastuzumab biosimilars, these differences need to be viewed in the context of the totality of evidence approach for biosimilarity, in which the clinical programme is a confirmatory step. In order to make informed decisions and to capture the potential of biosimilars, it is essential to provide oncologists with adequate information on the nature of the biosimilarity exercise and how the clinical development of a biosimilar is tailored to meet the licensing requirements.

## Supplementary information

MATERIAL Supplementary information-The arrival of trastuzumab biosimilars

## Data Availability

Not Applicable
