# Supplementary material for: The arrival of biosimilar monoclonal antibodies in oncology: clinical studies for trastuzumab biosimilars
Source: Br J Cancer. 2019 Jul 1;121(3):199–210. doi: 10.1038/s41416-019-0480-z (PMC6738325; doi:10.1038/s41416-019-0480-z)
Supplement: Supplementary file 1 — MATERIAL Supplementary information-The arrival of trastuzumab biosimilars [file 41416_2019_480_MOESM1_ESM.doc]

**Supplementary information**

Below, an overview is given of the phase III data for the different trastuzumab biosimilar(s) (candidates) that were derived from published scientific literature. Some of these data are not yet available in full text publication. The data and conclusions available from abstracts and posters should be considered as preliminary until published in a peer-reviewed journal or described in the European public assessment report of the respective medicine.

**Phase III clinical data per trastuzumab biosimilar (candidate)**

*ABP 980, Amgen/Allergan’s trastuzumab biosimilar (Kanjinti®)*

Amgen/Allergan compared its candidate, ABP 980, in a phase III study (“LILAC”) with the reference product (RP) in patients with HER2+ early breast cancer (EBC) (neoadjuvant and adjuvant setting, N=725).^1^ The primary endpoint of total pathological complete response (tpCR) was measured when the patient completed surgery after the neoadjuvant therapy phase.^1^ Results have been presented in abstract and full text.^1,2^ The equivalence margin was set at -13%, +13% with a 90% confidence interval (CI) for risk difference (RD) and at 0.759, 1.318 with a 90% CI for risk ratio (RR) of pCR. Secondary endpoints included safety. Based on predefined local review, the upper margin of the CI for both RD and RR of pCR slightly exceeded the equivalence margin (RD in pCR: 7.3% (90% CI: 1.2, 13.4), RR in pCR: 1.19 (90% CI: 1.033, 1.366)), excluding non-inferiority, but not potential superiority. Based on central independent review, RD and RR of pCR were 5.8% (90% CI: -0.5, 12.0) and 1.14 (90% CI: 0.993, 1.312) and the CI of both RD and RR were thus contained within the predefined equivalence margin.^1^ Adverse events (AEs) (≥1 AE: 80.2% vs 79.5% for ABP 980 and RP respectively) and grade ≥ 3 AEs (14.8% vs 14.1% for ABP 980 and RP respectively) were reported to be comparable between groups.^1^ The phase III trial of ABP 980 included a single switch in the adjuvant phase (results presented in abstract).^3^ Patients who received the RP (+ paclitaxel) during the neoadjuvant phase were randomized, while blinding was maintained, in two groups after surgery. A total of 171 patients switched from the RP to treatment with ABP 980 and 171 patients stayed on treatment with the RP in the adjuvant phase.^3^ At the time of primary analysis, 89 (52.0%) and 98 (57.3%) patients had an AE in the RP-RP continuing group and RP-ABP 980 switch group, respectively. Ten (5.8%) patients in each group had a grade ≥3 AE.^3^ One patient in the switch group developed binding, non-neutralizing anti-drug antibodies (ADAs) during the adjuvant phase. For one patient in each group cardiac failure was reported. The percentage of patients with disease progression, recurrence or death was 5.3% and 2.9% in the RP-RP continuing group and RP-ABP 980 switch group, respectively.^3^

*BCD-022, Biocad’s trastuzumab biosimilar candidate*

The results of the biosimilar candidate BCD­022 of the Russian manufacturer Biocad have been reported in abstract.^4^ A total of 126 patients with HER2+ metastatic breast cancer (MBC) were randomized to receive either BCD-022 or the RP in combination with paclitaxel every three weeks up to six cycles of therapy or until progression or intolerable toxicity. The primary endpoint was ORR. The reported ORR was 53.57% (95% CI 40.70-65.98%) in BCD-022 group and 53.70% (95% CI 40.60%-66.31%) in the RP group. The lower limit of the 95% CI for difference in ORR between groups (-19.83%) did not exceed the non-inferiority margin of -20%, showing that BCD-022 is non-inferior to the RP in terms of ORR in patients with HER2+ MBC.^4^ Other efficacy parameters, such as complete and partial response, stable disease and progression rate were reported to show similar results between treatment arms. No statistically significant differences in AEs, including serious AEs, were reported between groups. Neutralizing ADAs were detected in one patient in each group.^4^ Cardiovascular events were reported and included tachycardia (34.92 vs 19.67%), arterial hypertension (20.63 vs 18.03%) atrial fibrillation (0 vs 3.28%), extrasystoles (0 vs 1.64%) and aggravated myocardiodystrophy (1.59 vs 0%).^4^ BCD-022 is on the market in Russia, however it is highly unlikely, based on the trial parameters and clinical trial results, that BCD-022 would be accepted by a stringent regulatory authority such as the European Medicines Agency (EMA) or the US Food and Drug Administration (FDA). BCD-022 can thus not be considered as a biosimilar, before being evaluated in such a way. Biocad has not submitted a dossier for marketing authorization (MA) in Europe or the US.

*CT-P6, Celltrion’s trastuzumab biosimilar (Herzuma®)*

The results of CT-P6, the trastuzumab biosimilar Celltrion, in neoadjuvant setting have been reported in full text.^5^ Almost 550 patients were randomized in a double-blind manner to receive the biosimilar or the RP in combination with docetaxel and 5-fluorouracil, epirubicin and cyclophosphamide. The primary endpoint was tpCR after neoadjuvant therapy at surgery and secondary endpoints were ORR, PK, PD and safety. After surgery, patients received CT-P6 or the RP up to a total of one year treatment. The reported tpCR rate was 46.8% in CT-P6 and 50.4% in the RP. The 95% CIs for the estimate of treatment difference were within the equivalence margin (-0.15, 0.15) in both per protocol set (PPS) and intention-to-treat (ITT). The proportion of patients with at least one serious treatment related adverse event was 7% in the CT-P6 vs 8% in the RP group. Treatment emergent adverse events (TEAEs) due to heart failure were reported in 2% vs 1% for CT-P6 and the RP group, respectively. Of these, one patient from the RP withdrew treatment due to a significant left ventricular ejection fraction (LVEF) decrease.^5^ In addition to the phase III trial in EBC, a double-blind, randomized, phase III equivalence study to demonstrate equivalent efficacy and comparable safety of CT-P6 and the RP, both in combination with paclitaxel, as first-line treatment in patients with HER2+ MBC has been conducted.^6^ Results of this trial have been published in abstract and poster. A total of 475 patients with HER2+ MBC were randomized to receive either CT-P6 plus paclitaxel or the RP plus paclitaxel. The primary endpoint was ORR (complete or partial response) at six months. ORR was 57% for the CT-P6 group and 62% for the RP group during the first eight cycles of treatment. The difference in ORR was reported to be 5% (95% CI: -0.14, 0.04). The limits of the 95% CI for the difference in ORR were thus contained within the pre-defined equivalence margin of -0.15, 0.15.^6^ Authors concluded that equivalence between the biosimilar candidate and the RP has been observed for ORR in patients with HER2+ MBC in combination with paclitaxel as first-line therapy. AEs were comparable between groups. Cardiotoxicity was observed in eight (3.3%) vs 10 (4.3%) in the CT-P6 and the RP group respectively. No information on immunogenicity was reported so far.^6^

*Myl1401O – Mylan’s trastuzumab biosimilar (Ogivri®)*

The results of the phase III HERITAGE study of the trastuzumab biosimilar of Mylan, Myl1401O, have been published in full text. HERITAGE, a double blind, randomized comparative clinical trial to evaluate comparative efficacy and safety of Myl1401O with the RP, was performed in HER2+ MBC.^7^ A total of 500 patients were randomized to receive either Myl1401O or the RP with docetaxel or paclitaxel for a minimum of eight cycles (24 weeks). After completing eight cycles, Myl1401O or the RP was continued as single treatment until disease progression, unacceptable toxic effects, or death. The primary endpoint was ORR (complete or partial response) at week 24. EMA requested to use the difference in ORRs as primary efficacy analysis, with a two- sided 95% CI. The FDA advised to perform the primary efficacy analysis based on the ratio of ORR with a two-sided 90% CI. Equivalence was demonstrated if the CI fell entirely within the pre-defined equivalence range of −15% to +15%.

At week 24, ORR was 69.6% for Myl14010 compared to 64% for the RP. The difference in ORR was 5.53 (95% CI, −3.08 to 14.04). This 95% CI fell within the predefined equivalence boundaries of −15% and 15%. The ratio of ORR was 1.09. Both 90% CI (0.974-1.211) and exploratory 95% CI (0.954-1.237) were within the predefined equivalence margin.^7^ Secondary endpoints included time to tumour progression (TTP), progression-free survival (PFS) and overall survival (OS) at week 48. No statistically significant differences in TTP, PFS or OS were observed between the biosimilar candidate and the RP.^8^ Secondary endpoints evaluated at both 24 and 48 weeks included AEs, LVEF, and immunogenicity. Safety was comparable between the biosimilar candidate and the RP. The overall incidence of patients with at least one TEAE was 96.8% in the biosimilar group and 94.7% in the RP group. The majority of events were mild or moderate in severity in both treatment groups. The overall incidence of patients with at least one serious AE was 38.1% vs 36.2%, for MYL1401O and the RP respectively. There was no significant change in cardiac function from baseline to week 24 reported in either group. Further, immunogenicity was low and comparable between groups. Authors concluded that the Myl1401O trastuzumab biosimilar candidate was equivalent to the RP, in combination with a taxane as first line therapy for MBC, as measured by ORR at week 24.^7^

*PF-05280014 – Pfizer’s trastuzumab biosimilar (Trazimera®)*

Pfizer conducted a phase III trial comparing PF-05280014 with the RP (“REFLECTIONS B3271002” trial), both in combination with paclitaxel. The trial was conducted with patients with HER2+ MBC in first line treatment (N=707) with ORR by week 25 (and confirmed by week 30) as primary efficacy endpoint.^9^ The risk ratio for ORR was 0.940. The 95% confidence interval (0.842, 1.049) was within the pre-specified equivalence margin of (0.8, 1.25). The one year PFS (56% for PF-05280014 vs 52% for EU-RP) and one year survival (88.84% vs 87.96%) were similar between groups. No new safety signals were identified. The safety profile, including incidence of serious AEs, was further reported to be similar in both arms. No ADAs were detected, except for one patient treated with the RP. Up to cycle 5 day 8, mean trough and peak serum concentrations were similar for both agents.^9^ A separate, comparative, non-inferiority randomized controlled trial (“REFLECTIONS B3271004”) investigating similarity between the candidate and the RP was performed in 226 patients with EBC in neoadjuvant setting.^10^ The study was powered to test whether the candidate was non inferior (with a non-inferiority margin of -12.5%) to the EU-RP in the percentage of patients with cycle 5 C_trough_ >20 μg/mL. Efficacy was measured by the percentage of patients that achieved pCR and ORR. The percentage of patients with cycle 5 C_trough_ >20 μg/mL was 92.1% for PF-05280014 vs 93.3% for the EU-RP. The lower limit of the 95% CI (-8.02%, 6.49%) for the stratified difference between groups fell above the pre-specified non-inferiority margin; showing non-inferiority. The pCR rate was 47.0% (95% CI: 36.9%, 57.2%) for PF-05280014 vs 50.0% (95% CI: 39.0%, 61.0%) for the EU-RP. Central radiology review-assessed ORR was 88.1% (95% CI: 80.2%, 93.7%) for PF-05280014 vs 82.0% (95% CI: 72.5%, 89.4%) for the EU-RP. Grade 3-4 TEAEs were reported by 38.1% vs 45.5% of patients, for the candidate and the RP respectively. Only one patient (in the RP group) had a positive ADA titer.^10,11^

*SB3 – Samsung Bioepis’ trastuzumab biosimilar (Ontruzant®)*

The trastuzumab biosimilar candidate SB3 of Samsung Bioepis and the RP have been comparatively tested in a randomized, double-blind phase III trial in 800 patients treated with neoadjuvant therapy for HER2+ EBC.^12^ Results have been presented in full text.^12,13^ SB3 or RP were given for eight cycles concurrently with chemotherapy (four cycles docetaxel followed by four cycles 5-fluorouracil/epirubicin/cyclophosphamide). Subsequently, patients underwent surgery followed by ten cycles of adjuvant SB3 or RP. Equivalence was demonstrated if the 95% CI of the ratio or the 95% CI of the difference of the breast pathologic complete response (bpCR) rates were contained within the pre-defined equivalence margins (0.785, 1.546) and (-13%, 13%), respectively. The bpCR rates were 51.7% for SB3 and 42.0% for the RP. The ratio of bpCR and its 95% CI fell within the pre-defined equivalence margin (1.259 CI: 1.085, 1.460). For the RD of bpCR rate (10.70%, CI: 4.13, 17.26), the lower margin of the 95% CI was contained within the predefined equivalence margin, while the upper margin of the 95% CI fell outside the pre-defined equivalence margin – excluding non-inferiority but not potential superiority to the RP. Although, SB3 demonstrated its equivalence based on the ratio of bpCR rates, numerically, the bcPCR rate of the SB3 group was 10% higher compared to the RP group. A potential explanation can be found in the identified drift in the antibody-dependent cell-mediated cytotoxicity (ADCC)-related quality attributes of various batches of the RP.^14,15^ Seen that some of these batches were used for the RP group during the clinical testing, authors indicated that it could not be excluded that these shifts in quality attributes of the RP did not potentially impact the phase III similarity results.^14^ Secondary endpoints included tpCR, ORR, event-free survival (EFS), PK, immunogenicity, and safety. These were reported to be comparable between SB3 and the RP; tpCR rate (45.8% vs 35.8%) and ORR (96.3% vs 91.2%). The incidence of TEAEs was 96.6% vs 95.2%, for SB3 and the RP. The incidence of serious AEs was 10.5% vs 10.7% and ADA incidence 0.7% vs 0.0% for SB3 and the RP respectively.^14^ The one-year results included results on safety, immunogenicity, EFS and OS. The incidence of TEAEs was reported to be comparable between arms (97.5% vs 96.1% for SB3 and RP). Grade ≥3 AE were 74.3% and 71.9% for SB3 and RP respectively. EFS rates were 92.2% vs 91.6% in SB3 and RP (hazard ratio 0.94; 95% CI, 0.59 to 1.51). Three patients (0.7%) were ADA positive in each arm, confirming low and comparable immunogenicity. There were six deaths reported; one in the SB3 group, five in the RP group.^16^

# **References**

1 von Minckwitz G. Efficacy and safety of biosimilar ABP 980 compared with trastuzumab in HER2 positive early breast cancer. *Ann Oncol* 2017; **28**: 44.

2 von Minckwitz G Von, Colleoni M, Kolberg H, Morales S, Santi P, Tomasevic Z *et al.* Efficacy and safety of ABP 980 compared with reference trastuzumab in women with HER2-positive early breast cancer ( LILAC study ): a randomised , double-blind , phase 3 trial. *Lancet Oncol*; **19**: 987–998.

3 von Minckwitz G. Biosimilar ABP 980 in patients with early breast cancer: Results of single switch from trastuzumab to ABP 980. *Cancer Res* 2018; **78**: (4 Suppl):Abstract P5-20-13.

4 Shustova M, Burdaeva O, Alexeev S, Shelepen K, Khorinko A, Mukhametshina G *et al.* Efficacy and safety of BCD-022, trastuzumab biosimilar candidate, compared to herceptin: Results of international multicenter randomized double blind study in patients with HER2+ mBC. *Ann Oncol* 2016; **27**: Supplement 6: vi68–vi99.

5 Stebbing J, Baranau Y, Baryash V, Manikhas A, Moiseyenko V, Dzagnidze G *et al.* CT-P6 compared with reference trastuzumab for HER2-positive breast cancer: a randomised, double-blind, active-controlled, phase 3 equivalence trial. *Lancet Oncol* 2017; **18**: 917–928.

6 Im Y-H, Odarchenko P, Grecea D, Komov D, Anatoliy C V, Gupta S *et al.* Double-blind, randomized, parallel group, phase III study to demonstrate equivalent efficacy and comparable safety of CT-P6 and trastuzumab, both in combination with paclitaxel, in patients with metastatic breast cancer (MBC) as first-line treatment. *J Clin Oncol* 2013; **31**: 15 SUPPL. 1.

7 Rugo HS, Barve A, Waller CF, Bronchud MH, Herson J, Yuan J *et al.* Heritage: A phase III safety and efficacy trial of the proposed trastuzumab biosimilar Myl1401O versus Herceptin. *J Clin Oncol* 2016; **34**.

8 Rugo HS, Barve A, Waller CF, Hernandez-Bronchud M, Herson J, Yuan J *et al.* Effect of a Proposed Trastuzumab Biosimilar Compared With Trastuzumab on Overall Response Rate in Patients With ERBB2 (HER2)–Positive Metastatic Breast Cancer. *JAMA - J Am Med Assoc* 2017; **317**: 37–47.

9 Pegram M, Tan-Chiu E, Freyman A, Vana A, Hilton F, Zacharchuk C *et al.* A randomized, double-blind study of PF-05280014 (a potential trastuzumab biosimilar) vs trastuzumab, both in combination with paclitaxel, as first-line treatment for HER2-positive metastatic breast cancer. *Ann Oncol* 2017; **28**: v74–v108.

10 Lammers PE, Dank M, Masetti R, Abbas R, Hilton F, Coppola J *et al.* Neoadjuvant PF-05280014 (a potential trastuzumab biosimilar) versus trastuzumab for operable HER2+ breast cancer. *Br J Cancer* 2018; **119**: 266–273.

11 Lammers PE. A randomized, double-blind study of PF-05280014 (a potential biosimilar) vs trastuzumab, both given with docetaxel (D) and carboplatin (C) as neoadjuvant treatment for operable human epidermal growth factor receptor 2-positive (HER2+) breast cancer. *Ann Oncol* 2017; **28**: 45.

12 Pivot X, Bondarenko I, Nowecki Z, Dvorkin M, Trishkina E, Ahn J-H *et al.* Phase III, Randomized, Double-Blind Study Comparing the Efficacy, Safety, and Immunogenicity of SB3 (Trastuzumab Biosimilar) and Reference Trastuzumab in Patients Treated With Neoadjuvant Therapy for Human Epidermal Growth Factor Receptor 2–Positive Early. *J Clin Oncol* 2018; **36**: 968–974.

13 Pivot X, Bondarenko I, Nowecki Z, Dvorkin M, Trishkina E, Ahn JH *et al.* A phase III study comparing SB3 (a proposed trastuzumab biosimilar) and trastuzumab reference product in HER2-positive early breast cancer treated with neoadjuvant-adjuvant treatment: Final safety, immunogenicity and survival results. *Eur J Cancer* 2018; **93**: 19–27.

14 Pivot X. A randomized, double-blind, phase III study comparing SB3 (trastuzumab biosimilar) with originator trastuzumab in patients treated by neoadjuvant therapy for HER2-positive early breast cancer. 2017 ASCO Annu. Meet. 2017; : Abstract 509.

15 Kim S, Song J, Park S, Ham S, Paek K, Kang M *et al.* Drifts in ADCC-related quality attributes of Herceptin®: Impact on development of a trastuzumab biosimilar. *MAbs* 2017; **9**: 704–714.

16 Pivot X. One-year safety, immunogenicity, and survival results from a phase III study comparing SB3 (a proposed trastuzumab biosimilar) and originator trastuzumab in HER2-positive early breast cancer treated with neo-adjuvant-adjuvant treatment. *Ann Oncol* 2017; **28**: 45.
